# Intragenic *DOK7* deletion detected by whole-genome sequencing in congenital myasthenic syndromes

**DOI:** 10.1212/NXG.0000000000000152

**Published:** 2017-05-03

**Authors:** Yoshiteru Azuma, Ana Töpf, Teresinha Evangelista, Paulo José Lorenzoni, Andreas Roos, Pedro Viana, Hidehito Inagaki, Hiroki Kurahashi, Hanns Lochmüller

**Affiliations:** From the Institute of Genetic Medicine (Y.A., A.T., T.E., P.J.L., A.R., H.L.), Newcastle University, UK; Division of Neurology (P.J.L.), Federal University of Parana, Brazil; Leibniz-Institut für Analytische Wissenschaften ISAS e.V. (A.R.), Germany; Department of Neurosciences and Mental Health (P.V.), University of Lisbon, Portugal; and Division of Molecular Genetics (H.I., H.K.), Fujita Health University, Japan.

## Abstract

**Objective:**

To identify the genetic cause in a patient affected by ptosis and exercise-induced muscle weakness and diagnosed with congenital myasthenic syndromes (CMS) using whole-genome sequencing (WGS).

**Methods:**

Candidate gene screening and WGS analysis were performed in the case. Allele-specific PCR was subsequently performed to confirm the copy number variation (CNV) that was suspected from the WGS results.

**Results:**

In addition to the previously reported frameshift mutation c.1124_1127dup, an intragenic 6,261 bp deletion spanning from the 5′ untranslated region to intron 2 of the *DOK7* gene was identified by WGS in the patient with CMS. The heterozygous deletion was suspected based on reduced coverage on WGS and confirmed by allele-specific PCR. The breakpoints had microhomology and an inverted repeat, which may have led to the development of the deletion during DNA replication.

**Conclusions:**

We report a CMS case with identification of the breakpoints of the intragenic *DOK7* deletion using WGS analysis. This case illustrates that CNVs undetected by Sanger sequencing may be identified by WGS and highlights their relevance in the molecular diagnosis of a treatable neurologic condition such as CMS.

Congenital myasthenic syndromes (CMS) are inherited disorders characterized by fatigable muscle weakness with or without other associated signs or symptoms.^1^ They are caused by mutations in genes expressed at the neuromuscular junction (NMJ). DOK7 is one of the components of the NMJ and an activator of the muscle-specific tyrosine kinase (MuSK).^2^ Recessive mutations in *DOK7* cause approximately 10% of the genetically diagnosed CMS cases.^1^

CMS are heterogeneous diseases, and to date, more than 25 genes have been reported to be causative. Consecutive single-gene screening has been routinely used as a diagnostic tool; however, next-generation sequencing allows the analysis of all these genes simultaneously to identify the causative variant and obtain a genetic diagnosis. The efficacy of whole-exome sequencing (WES) for the diagnosis of CMS cases has been reported,^3,4^ as well as its ability to identify new causal genes.^5,6^ However, the limitation is that WES is designed to detect only protein-coding regions and exon-intron boundaries of the genome.

On the other hand, whole-genome sequencing (WGS) allows the analysis of deep intronic, intergenic, and other noncoding regions. Furthermore, WGS allows to detect copy number variations (CNVs), as coverage is more homogeneous than that of WES.^7^

We present a CMS case in which a large intragenic *DOK7* deletion was identified by WGS compound heterozygous to a known exonic mutation.

## METHODS

### *DOK7* screening.

DNA from the patient was extracted from whole blood by standard methods. Screening of hot-spot mutations was performed by Sanger sequencing, encompassing a region of ∼600 bp covering the previously reported European founder mutation c.1124_1127dup.^2^ Subsequently, full screening of coding regions and exon-intron boundaries of the *DOK7* gene was performed. Primer sequences are listed in table e-1 at Neurology.org/ng. Annotation of the human *DOK7* cDNA is according to the GenBank accession number NM_173660.

### Mutation analysis by WGS.

WGS was performed by the TruSeq PCR–free library preparation kit and HiSeqX v2 SBS kit (Illumina, San Diego, CA) for 30× mean coverage on a HiSeqX sequencer. Reads were mapped against hg19 reference genome using the Burrows-Wheeler transform,^8^ and duplicates were removed using Picard tools.^9^

Sequence variants were called using the Genome Analysis Toolkit.^10^ WGS data were then analyzed using deCODE's platform (Clinical Sequence Miner; WuXi NextCODE, Cambridge, MA). Rare variants were filtered by threshold of coverage (≥8), variant call (≥2), and ratio of variant (≥0.2) and allele frequency of 1% in 1000 Genomes database.^11^

### Sanger sequencing of large deletion.

We amplified DNA samples to identify the suspected intragenic deletion with primers 5′-CCCAGATGGTGCGCTTGCTCC-3′and 5′-GCCCACCCCCTCACGCTCAG-3′. The PCR protocol comprised 35 cycles and annealing temperature of 68°C using HotStarTaq DNA polymerase with Q-Solution for the GC rich region (QIAGEN, Düsseldorf, Germany).

### Standard protocol approvals, registrations, and patient consents.

All human studies including genetic analysis were approved by institutional review boards, and appropriate written informed consent was obtained from all the patients and family members.

## RESULTS

### Clinical findings.

The patient is a 39-year-old Portuguese man who presented with bilateral ptosis and exercise-induced muscle weakness. He had no family history of muscle disease, and his motor milestones in childhood were normal. He showed mild ptosis from infancy and noticed mild lower limb weakness at 13 years of age. He was admitted to hospital for a month because of sudden severe generalized muscle weakness and worsening ptosis at 15 years of age. He has bilateral facial weakness and winged scapula, and the clinical diagnosis of a neuromuscular transmission defect was confirmed by neurophysiologic studies. EMG showed myopathic changes on facial muscles. Repetitive nerve stimulation showed a remarkable decremental response of 76% in proximal muscles. Both antiacetylcholine-receptor and anti-MuSK antibodies were negative, and immunosuppressive treatment was unsuccessful. Acetylcholinesterase (AChE) inhibitor of pyridostigmine up to 360 mg/d for 10 years had little effect and was discontinued without clinical deterioration after the trial of oral administration of salbutamol which effected significantly. He has not experienced severe muscle weakness for 5 years since salbutamol was started.

### *DOK7* screening.

Based on the limb-girdle clinical presentation of the patient, a hot-spot region of *DOK7* was investigated as a first screening step. Sanger sequencing revealed that the patient carried the heterozygous c.1124_1127dup reported as a founder mutation in European CMS patients.^2^ This mutation was not present in the mother (DNA from the father was unavailable). However, this single heterozygous mutation does not explain *DOK7*-CMS, which invariably shows autosomal recessive inheritance. To identify a second heteroallelic *DOK7* variant, the whole coding region and exon-intron boundaries of the *DOK7* gene were Sanger sequenced, but no potentially pathogenic exonic or splice site variants were found. The sample was therefore subjected to WGS to try to identify other mutations within the *DOK7* gene or elsewhere in the genome.

### WGS analysis.

As expected, applying a standard pipeline for variant filtering (minor allele frequency 1% in coding region), the heterozygous c.1124_1127dup in *DOK7* was detected in the WGS data. This filtering did not identify any other coding variants in known CMS causal genes.

**Figure 1:**
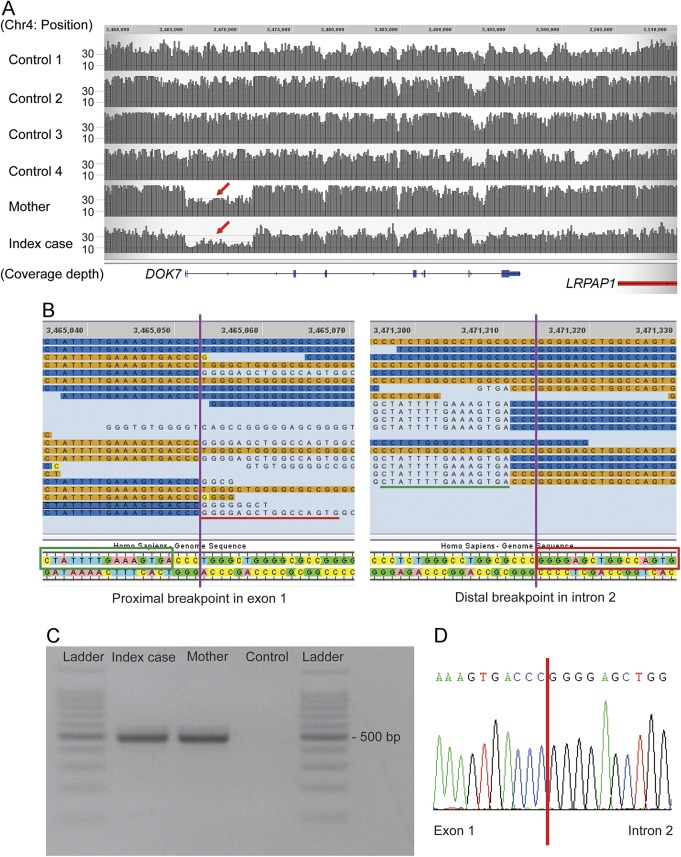
Whole-genome sequencing analysis and allele-specific PCR (A) Both index case and his mother show reduced read depth (coverage) from exon 1 to deep intron 2 of the *DOK7* gene (red arrow). Controls 1–4 correspond to samples sequenced and analyzed through the same pipeline and without the diagnosis of congenital myasthenic syndromes. (B) Split reads were observed at both presumed breakpoints. Nucleotides matching the reference sequence of *DOK7* are highlighted in orange/blue. Single unmatched nucleotides are highlighted in yellow, and further unmatched sequences are not highlighted. The unmatched sequence (indicated with red/green underline) of the split reads of the proximal breakpoint aligns to the reference sequence (indicated in green/red boxes) at the distal breakpoint, and vice versa. (C) The expected products amplified by allele-specific PCR were identified in the index case and the mother. (D) The junction of the breakpoint in the allele with the intragenic deletion was confirmed by Sanger sequencing of the PCR product. Coverage and reads were drawn by the graphical user interface of Sequence Miner 5.21.1 (WuXi NextCODE).

However, visual inspection of the sequencing reads of the *DOK7* gene for this patient revealed that the read depth for exons 1 and 2 was lower than that of neighboring regions and other control samples (figure 1A). Furthermore, there were no heterozygous variants within this region, indicating a run of homozygosity or hemizygosity suggesting a single copy region. Close inspection of the boundaries of this region showed that in some instances, sections of the sequencing reads did not match the reference sequence. These reads were considered chimeric or split reads, as the unmatched sequences did align to a different region of the genome. Split reads are indicative of structural variation. In fact, the 3′ section of the split reads of the proximal boundary aligns to the 3′end of the distal boundary, and vice versa (figure 1B, red underline and red box). The proximal and distal breakpoints lie approximately 6 kb away. These findings suggested that this patient has a heterozygous 6-kb deletion in *DOK7* encompassing exons 1 and 2.

### Identification and analysis of the intragenic *DOK7* deletion.

We performed PCR using a pair of primers designed around 250 bp away from the presumed breakpoints of the deletion, between the 5′ untranslated region and intron 2. The expected product of 488 bp was amplified in the DNA samples of the patient, but not in control DNA (figure 1C). The junction of the 2 breakpoints was identified by Sanger sequencing of the PCR product (figure 1D). The exact size of the deletion is 6,261 bp. The deletion was also detected by PCR in the mother, who did not carry the c.1124_1127dup mutation. We therefore concluded that the CMS in the patient is caused by the compound heterozygous mutations in *DOK7*.

**Figure 2:**
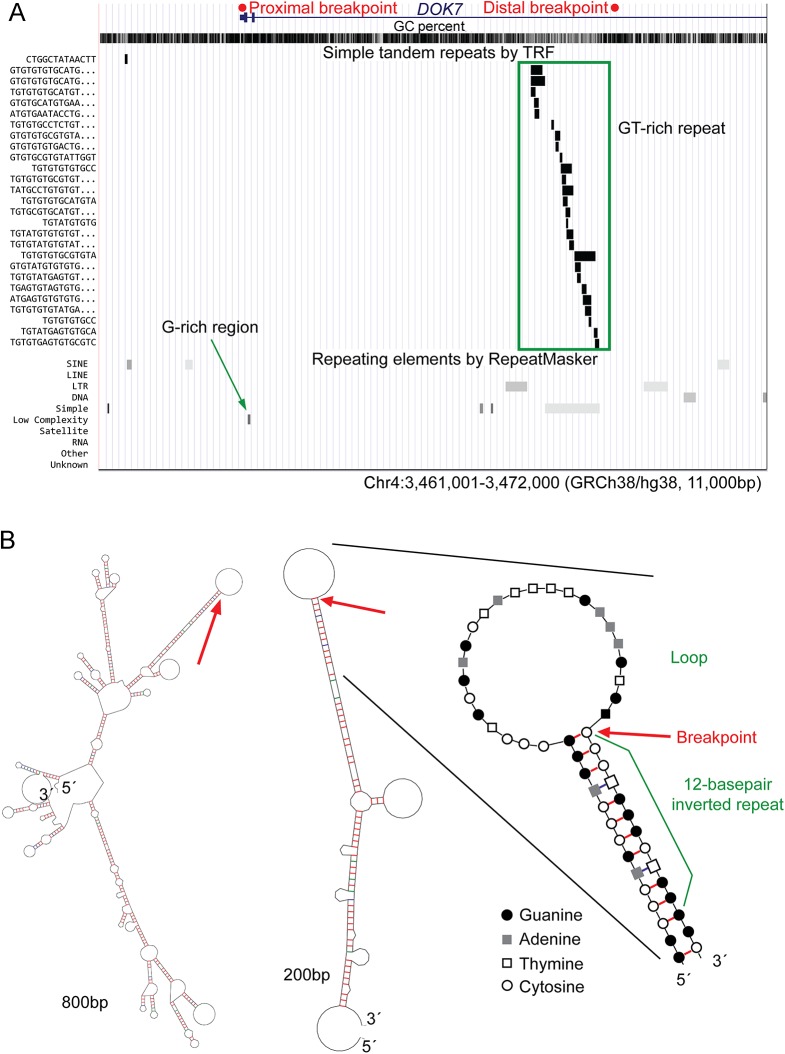
Analysis of the breakpoints of the intragenic 6-kb deletion (A) University of California Santa Cruz genome browser (genome.ucsc.edu/) view of the deleted region showing the Simple Tandem Repeats track (based on Tandem Repeats Finder, TRF^18^) and the Repeating Elements track (based on RepeatMasker^19^). GT-rich repeat regions (green box) are seen around the distal breakpoint, and a G-rich region (green arrow) is located near the proximal breakpoint. (B) The secondary DNA structure with the lowest delta G value was predicted by the mfold tool (unafold.rna.albany.edu/?q=mfold) for the 800 and 200 bp regions around the proximal breakpoint. An enlarged view of the breakpoint area highlighting the complementary nucleotides is also shown. The proximal breakpoint (indicated by the red arrows) is at the boundary of a loop and a 12-bp inverted repeat that may cause stalling of DNA replication. It is possible that deletion/duplication can occur if stalled replication resumes using an alternate location on the same chromosome. Red/blue/green bars represent hydrogen bonds between G-C/T-A/G-T.

The 2 breakpoints of the deletion have a C-triplet homology region, and the deleted region contains a G-rich region and GT-rich repeat region (figure 2A). In silico secondary structure analysis using the prediction program mfold^12^ showed that the proximal breakpoint is at the boundary of a loop and a 12-bp inverted repeat (figure 2B). This may cause stalling of DNA replication and subsequently result in chromosomal structural changes including deletions, if replication resumes using an alternate chromosomal location.

### Screening of the intragenic deletion in a CMS cohort.

To identify carriers of single heterozygous mutations in *DOK7* (i.e., without a second rare variant within coding regions and exon-intron boundaries), we interrogated our database of clinically diagnosed CMS cases referred to us in the years 1996–2015. The total number of patients with CMS was 577, of which 7 genetically unsolved cases had single frameshift mutations in *DOK7* (c.1124_1127dup in 6 cases and c.1378dup in 1 case). These samples were amplified using the deletion-specific pair of primers used to detect the 6-kb deletion of the index family. All 7 samples were negative using this PCR method. This does not exclude that they carry CNVs in *DOK7* different from the one described in this study.

## DISCUSSION

We identified an intragenic *DOK7* deletion in a patient with clinically diagnosed CMS. Patients lacking a second heteroallelic mutation in *DOK7* were reported in a previous study.^2^ Moreover, multiexon genomic deletions of *RAPSN*^13^ and *COLQ*^14^ have also been identified as causative of CMS. It is therefore conceivable that CNVs in *DOK7* may explain a proportion of cases assessed as negative or inconclusive by conventional sequencing analysis.

Our study shows the advantage of WGS analysis and detailed interrogation for detecting CNVs, using coverage and visual analysis of split reads. Traditionally, multiplex ligation–dependent probe amplification (MLPA) is considered the method of choice to detect previously described CNVs, where kits are available commercially. To identify new CNVs, however, specific MLPA primers for each gene need to be designed, rendering it expensive and time consuming for testing a genetically heterogeneous syndrome such as CMS. Array comparative genomic hybridization (aCGH) is also a valuable method for CNVs analysis; nevertheless, deletions/duplications are not detectable by aCGH if they are shorter than the spacing of the hybridization probes. In addition, neither MLPA nor aCGH can detect single nucleotide variants. Despite WES being widely used for clinical sequencing, the library preparation step results in uneven coverage, which makes the estimation of CNVs by read depth less reliable. This can be overcome by the homogenous coverage of WGS, allowing both the detection of single nucleotide as well as CNV.

WGS analysis is still more expensive than WES and Sanger sequencing. In addition, computational tools need further improvement in sensitivity and specificity to detect CNVs exhaustively.^15^ Taken together, we believe that WGS is advantageous and will become the method of choice for genetic diagnosis in rare, heterogeneous conditions such as CMS. We suggest that previously unsolved cases or the carriers of a single mutation in a causal gene are especially suitable cases of CMS for WGS analysis. The 6-kb deletion was not identified in other cases tested by PCR, although it is inherited from the mother, suggesting this is likely a private mutation. However, it is possible that other CNVs in *DOK7* underlie in CMS cases.

We also determined the breakpoints of the 6-kb deletion, and analysis of the sequence and secondary structure suggested that long inverted repeats might cause the development of the deletion due to a stall of replication, and microhomology might have played a role in the repair process.^16^ Further documentation of breakpoints and sequences would help understand the mechanism for the development of CNVs.

Obtaining genetic diagnosis of CMS is very important because the therapy varies depending on the affected gene. Poor response to AChE inhibitors is often observed in patients affected by limb-girdle CMS due to *DOK7* mutations. Salbutamol therapy has now been started for the patient described in this study, which has been reported of good response in *DOK7*-CMS.^17^

## Supplementary Material

Data Supplement

## GLOSSARY

aCGH: array comparative genomic hybridization
AChE: acetylcholinesterase
CMS: congenital myasthenic syndromes
CNV: copy number variation
MLPA: multiplex ligation–dependent probe amplification
MuSK: muscle-specific tyrosine kinase
NMJ: neuromuscular junction
WES: whole-exome sequencing
WGS: whole-genome sequencing
