# Supplementary material for: Intragenic DOK7 deletion detected by whole-genome sequencing in congenital myasthenic syndromes
Source: Neurol Genet. 2017 May 3;3(3):e152. doi: 10.1212/NXG.0000000000000152 (PMC5415388; doi:10.1212/NXG.0000000000000152)
Supplement: Data Supplement [file supp_3_3_e152__index.html]

Data Supplement 

# Intragenic *DOK7* deletion detected by whole-genome sequencing in congenital myasthenic syndromes

## Data Supplement

**Files in this Data Supplement:**

- Table\_e-1.docx
