# Supplementary material for: Intragenic DOK7 deletion detected by whole-genome sequencing in congenital myasthenic syndromes
Source: Neurol Genet. 2017 May 3;3(3):e152. doi: 10.1212/NXG.0000000000000152 (PMC5415388; doi:10.1212/NXG.0000000000000152)
Supplement: Data Supplement [file supp_3.3.e152_Table_e-1.docx]

| exon | forward | reverse |
| --- | --- | --- |
| ex1 | 5′- cgcgggacggtggggccaga -3′ | 5′- cccccgcgcccccgacg -3′ |
| ex2 | 5′- ggctcacgctcccccctgt -3′ | 5′- gggccctgctctgagcgtt -3′ |
| ex3 | 5′- cagcccgggtctctgcactgtcacg -3′ | 5′- catgaatgtcccatcttcctgcacg -3′ |
| ex4 | 5′- ctgttgcctcctctcatgat -3′ | 5′- agaggaccttggacatcgag -3′ |
| ex5 | 5′- gtcggctcttggtggagtttgctg -3′ | 5′- atctaactggggctgacaaatcac -3′ |
| ex6 | 5′- cacagagggggataaccactgagtc -3′ | 5′- tgcagcccccacacatgcatggatg -3′ |
| ex7-1 | 5′- ctggaaggggtggggagcga -3′ | 5′- tgctgcccgcgtaggacgaga -3′ |
| hotspot^a^ | 5′- cacagactggacatgccgaaaa -3′ | 5′- catggccagagcaatcctcgtc -3′ |
| ex7-2 | 5′- aacagtgcggccagggactca -3′ | 5′- agccccttcctccggtgg -3′ |

**Table e-1. Primers used for Sanger sequencing**

^a^The primer pair for hotspot covers c.1124_1127dup in exon7 (NM_173660). Three primer pairs (ex7-1, hotspot and ex7-2) are required to investigate the entire coding region of exon7.
